# Multiple-trait quantitative trait locus mapping with incomplete phenotypic data

**DOI:** 10.1186/1471-2156-9-82

**Published:** 2008-12-05

**Authors:** Zhigang Guo, James C Nelson

**Affiliations:** 1 Department of Plant Pathology, Kansas State University, Manhattan, Kansas, USA 66506; 2 Syngenta Seeds, Inc., Clinton, Illinois, 61727, USA

## Abstract

**Background:**

Conventional multiple-trait quantitative trait locus (QTL) mapping methods must discard cases (individuals) with incomplete phenotypic data, thereby sacrificing other phenotypic and genotypic information contained in the discarded cases. Under standard assumptions about the missing-data mechanism, it is possible to exploit these cases.

**Results:**

We present an expectation-maximization (EM) algorithm, derived for recombinant inbred and F_2 _genetic models but extensible to any mating design, that supports conventional hypothesis tests for QTL main effect, pleiotropy, and QTL-by-environment interaction in multiple-trait analyses with missing phenotypic data. We evaluate its performance by simulations and illustrate with a real-data example.

**Conclusion:**

The EM method affords improved QTL detection power and precision of QTL location and effect estimation in comparison with case deletion or imputation methods. It may be incorporated into any least-squares or likelihood-maximization QTL-mapping approach.

## Background

Statistical methods for identifying and mapping genes controlling complex traits, commonly known as quantitative trait loci or QTL, have been developed to a high degree. The primary focus has been on methods for single traits ([1-8] and many others). It was proposed [9,10] that multi-trait QTL mapping methods that consider simultaneously several correlated phenotypic traits, or a single trait measured in several environments, offer increased detection power and precision of location and effect estimation over single-trait QTL mapping. This is because trait-by-trait QTL-searching neglects information contained in the data about the common influence of a QTL on more than one trait, in more than one environment, or [11] at more than one developmental stage. Multi-trait (MT) QTL mapping allows a formal test of pleiotropy of a QTL for multiple traits or QTL-by-environment interaction for a single trait measured across multiple environments. The enhancement by MT of QTL-detection power is greatest when the QTL induces covariation between the tested traits in the direction opposite to that from "background" sources [12,13]. These advantages have been exploited in animal [14-16] and plant [17] studies.

With the promise of increased power from a multivariate approach comes an interesting problem: what to do when some of the multivariate data are missing.

Two main statistical approaches have been elaborated for multi-trait QTL analysis: regression [10,18-21] and maximum likelihood or ML [9]. Regression QTL-mapping methods, though easier to implement and faster to compute, give biased parameter estimates with sparse markers [22] or when QTLs interact or are closely linked [23], while ML methods are free of these defects [23]. It has also been proposed to transform multiple traits into canonical variates so that conventional univariate interval QTL mapping can be applied [18,24,25], but interpretation of the results is difficult.

Though QTL-mapping data are often incomplete, information-recovery methods are at present applied only to genotypic data. For incompletely informative marker-genotype data, posterior distributions are readily estimated from flanking markers in the same individual [26]. For unknown QTL genotypes at tested positions in map intervals, ML methods estimate posterior distributions simultaneously with the parameters of a phenotypic mixture distribution [4], while regression methods [1] replace missing QTL genotypes with their expectations given flanking markers. Variations based on sampling include multiple imputation (MI) as described by [27] and [22] and Bayesian approaches (*e.g*. [5-7,28,29]).

In contrast to genotypic data, missing phenotypic data for any trait results in discarding all cases (individuals) lacking even one value, sacrificing all other phenotypic and genotypic information available for these cases. The problem was recognized by [20], but they provided no solution, nor does conventional QTL-mapping software offer an alternative to this "casewise" [30] deletion. Is there one?

Completion of incomplete multivariate data may be done by imputation (single or multiple), by EM algorithm, or by Bayesian approaches. Single imputation typically replaces missing data with three kinds of values: a value drawn from a model-based distribution, a mean of other observations of the same variable, or a conditional mean calculated by least-squares regression on predictors. MI [31,32] fills in missing data by imputing multiple (*e.g*. 3–5) times to produce several complete datasets, with parameter estimates calculated as the average over the results from these datasets. The defect of imputation methods, in analyses such as QTL mapping where we want ML estimates of statistics, is that bias is introduced by maximization of the likelihood over both original and imputed data. In contrast, the EM algorithm as described by [33] focuses not on replacing a missing value with its expectation, but on using the information available in the original dataset. In the framework of EM, missing data imputed are in effect integrated out of the complete-data log likelihood by iterative refinement of their expectation. An EM method described by [34] addressed the problem of missing genotype or phenotype data in single-marker QTL analyses by the use of flanking-marker genotypes. While free of the dependence on recombination estimates to which interval-mapping methods are subject, the method accommodated only single traits. [35] provided an EM algorithm for incomplete multivariate data and extended it to accommodate multiple regression with missing responses. A Bayesian approach developed by [36] for joint mapping with multiple traits in outbred populations employed an identity-by-descent (IBD)-based variance-components model and reversible-jump Markov-chain Monte Carlo (MCMC) estimation, but did not consider missing phenotypic and genotypic data. It would be possible to derive an MCMC algorithm to sample missing phenotypic entries from their posterior distribution, though Bayesian approaches are computationally intensive and often criticized for lacking a test statistic.

Here we describe an adaptation of the EM method of [35] to the case of multi-trait QTL mapping with incomplete phenotypic data. For simplicity we have limited our scope of mating design to biparental crosses between inbreds. We show that the tests for QTL main effects may be constructed as in [9], and we describe the properties and behavior of the test statistics and QTL effect and position estimates based on simulation studies and a real example.

## Results

### Power

**Figure 1:**
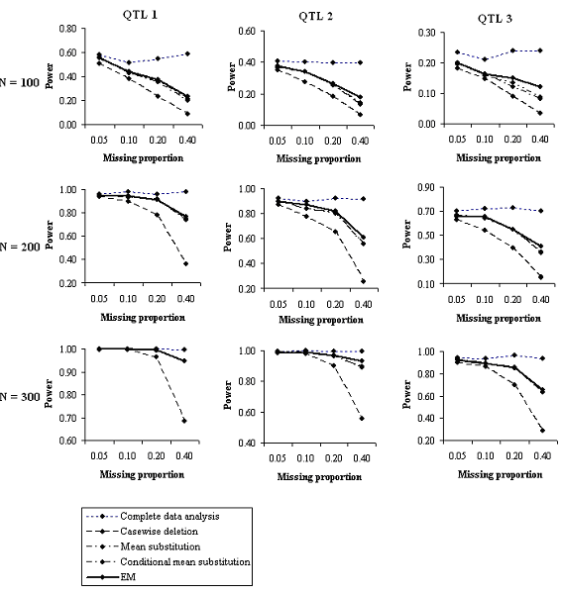
Statistical power of five multiple-trait QTL-mapping methods in simulated populations with four levels of missing data.

As expected, power was highest when data were complete (Figure 1). When data were missing, EM, MS and CMS gave power superior to CaD in all cases. MS and CMS gave similar power, equal to or lower than that of EM. The gain in power for EM over CaD increased with the proportion of missing data. This trend was also seen for gain in power of EM over MS or CMS, but to a lower degree.

**Figure 2:**
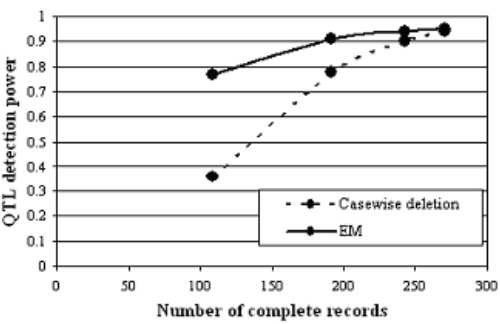
Power of QTL 1 detection after casewise deletion and by the EM method as a function of the number of complete trait records for 300 simulated RILs.

Figure 1 also shows that EM gave QTL detection power about equal to that supplied by CaD with half the proportion of missing data. Simple probability calculations yield the numbers to which this power relationship corresponds. As an example, in a population of size 300 with 0.4 of the data missing from each of two traits, the EM method was operating on only 108 lines carrying complete data and another 144 lines with partial data, but achieved power corresponding to 192 lines with complete data. The increase in effective (equivalent-power) number of complete records achieved by the EM method can be estimated graphically from Figure 2. Here the effective complete-data sample sizes achieved by EM were about 271, 255, 230, and 190, representing gains of 1, 12, 38 and 82 over the number of complete records available for CaD at missing levels of 0.05, 0.1, 0.2 and 0.4.

### Specificity

All the methods gave similar QTL-detection specificity of 0.98 to 1.00, except with sample size 100 and missing proportion 0.40, where CaD gave specificity as low as 0.93.

### Accuracy and precision of QTL effect estimation

**Figure 3:**
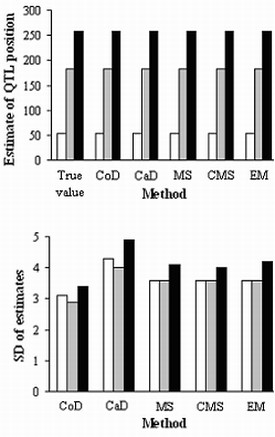
**QTL position estimates and standard deviations with 200 simulated RILs and 0.40 missing proportion for each trait**. White, gray, and black bars represent QTLs 1, 2 and 3. CoD: complete data analysis; CaD: casewise deletion; MS: mean substitution; CMS: conditional mean substitution; EM: EM algorithm; SD: standard deviation

**Figure 4:**
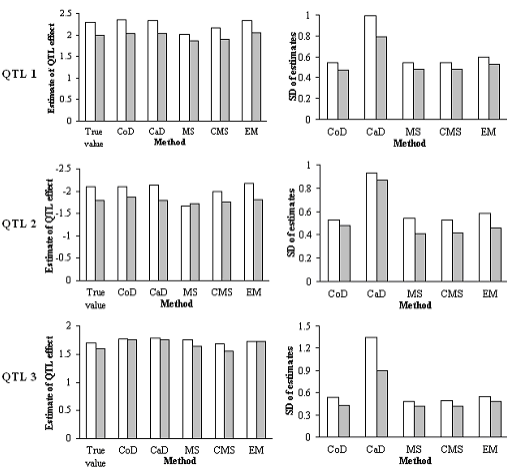
**QTL effect estimates and their standard deviations with 200 simulated RILs and 0.40 missing percentage for each trait**. White bars represent trait 1 and gray trait 2. CoD: complete data analysis; CaD: casewise deletion; MS: mean substitution; CMS: conditional mean substitution; EM: EM algorithm; SD: standard deviation

All methods gave reasonable estimates of QTL positions. CoD and CaD provided the highest and lowest precisions for QTL position estimation (Figure 3), while those of MS, MS, and EM were very similar and intermediate. For QTL effects (Figure 4), CoD, CaD and EM provided unbiased estimates, while both MS and CMS underestimated these parameters, CMS by slightly less. The extent of underestimation tended to increase with missing percentage and decrease with sample size (not shown here).

### Real data analyses

**Figure 5:**
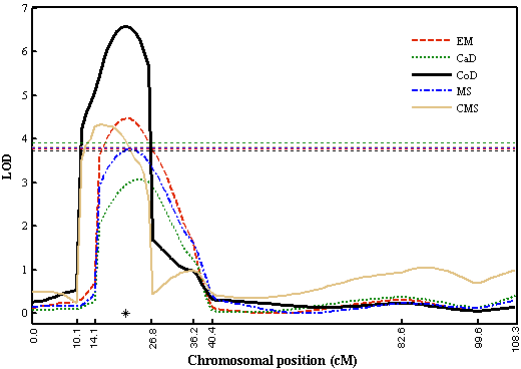
**LOD profiles of several approaches to analysis of a putative QTL for two traits in a 325-line doubled-haploid rice population with 10% simulated missing data**. CoD: complete data analysis; CaD: casewise deletion; MS: mean substitution; CMS: conditional mean substitution; EM: EM algorithm. Horizontal dotted lines in the corresponding colors show the empirical LOD thresholds for these methods. The asterisk marks the position (at 21 cM) of the QTL identified by CoD.

In the selected example from a real dataset, EM provided higher power for the detection of a QTL on rice chromosome 12 than CaD and MS (Figure 5). While CMS also identified the QTL, its position estimate was somewhat biased, as were those of CaD and MS (Table 1). In general, EM provided estimates of QTL position and effect closer to those from CoD than were the estimates from CaD, MS and CMS.

**Table 1:** QTL statistics for analysis of two traits in a 325-line doubled-haploid rice population with 10% simulated missing data, estimated by several approaches.

**Method**	**Position (cM)**	**LOD**	**Additive effect**	
			**Sheath blight**	**Heading days**
**EM**	21	4.47	-0.38	0.80
**CaD**	24	3.07	-0.33	0.70
**MS**	22	3.76	-0.32	0.66
**CMS**	16	4.41	-0.55	0.59
**CoD**	21	6.58	-0.44	1.06

**Key **EM: EM algorithm; CaD: casewise deletion; MS: mean substitution; CMS: conditional mean substitution; CoD: complete data analysis.

## Discussion

While any substitution of unavailable data by their expectations based on trends in available data will increase the precision of parameter estimates over those obtained by CaD, the EM-based multi-trait QTL mapping method we propose here is superior to MS and CMS for several reasons. MS underestimates phenotypic variation and QTL effect due to fill-in of missing data with a single value, resulting in decreased power compared with our method especially when amounts of missing data are relatively large. The same trend can be observed for CMS, which, as a precursor of the EM algorithm, is closely related to a single EM iteration [35]. Although CMS gave better estimates of QTL effect than MS, it still underestimates variance [35].

While we did not include MI [31,37] in the simulation study, we doubt its potential utility for multi-trait QTL mapping with missing trait data. We investigated MI by filling in missing trait data with values sampled from their conditional distributions under the null and alternative hypotheses given the observed trait values. Resulting logarithm-of-odds (LOD) profiles were sawtoothed (not shown here) due to random sampling, and a different profile could be obtained with each analysis even with many imputations (*e.g*. 100 compared with 3–5 in regular MI) performed at each QTL test position. For these reasons, apart from the high computational cost, we did not pursue this method further.

For MS, CMS, and even MI, the effects on QTL mapping of introducing imputed data need further study. Although simulation results showed specificities close to those of our method, complete-data analysis, and CaD, the bias imposed on the LOD test statistic by introduction of these "artificial" data remains unknown. Interestingly, in the real-data example we chose for illustration, CMS, besides apparently biasing the location of the QTL, gave higher background LOD scores than CoD in regions away from the QTL, while the EM and other approaches did not (Fig. 5). In fact, imputation of missing data is also performed in the E step of our EM algorithm. But this kind of imputation only furnishes a pivot to facilitate parameter estimation and is actually not involved in the likelihood calculation. Thus, theoretically, the EM-based method does not bias QTL detection and parameter estimation as may imputation methods.

The information gain of our method over CaD, MS, and CMS depends on the amount of missing trait data. The reason is readily explained by the following example for CaD. Consider a sample of 200 individuals with missing proportion 0.1 for each of two traits independently. The average number of individuals available for CaD is 162 and that for EM 198, and the difference is 36. This difference expands to 96 with a missing proportion of 0.4. In other words, power is lost more slowly with data loss when the information-recovering EM method is applied.

Cofactor markers too may lack genotype data. In our model, these are replaced by expectations given flanking markers, computed by the method of [26]. Prediction of missing trait data employs these imputed cofactor genotypes, with a resulting potential for error in parameter estimation. While such error could be minimized by extension of our method to include missing cofactor data, we suspect that the improvement would hardly justify the computation, and remark that the issue is shared by all composite interval-mapping methods.

While the EM method should give more power than the information-discarding alternatives regardless of the chosen QTL acceptance threshold, we do not know how to find the optimum threshold for multiple-trait mapping when our algorithm is applied. Indeed, the question has not to our knowledge been satisfactorily answered to date even for the case of complete data. Thresholds in our simulation experiment were based on analyses of simulated populations lacking QTLs but with complete trait data conforming to the same variance structure as the QTL population – an option not available to an analyst in practice. A working method might be to adopt a threshold lying between the overly conservative one calculated from permutation (of individual records for all traits) applied to the complete data remaining after CaD and the insufficiently conservative one that would be obtained from permutation applied under our reconstruction algorithm. For nonpathological data sets our simulation results suggest that this range will be relatively narrow. The quick and approximate method of [38] might serve as well as any for establishing thresholds.

Some extensions of the EM method are promising. First, we have derived the EM calculation of the hypothesis test for QTL main effect. By following the procedure of [9], one may derive specific EM implementations for other hypothesis tests including for QTL-by-environment interaction, pleiotropy, and pleiotropy vs. close linkage. Second, the EM method may be extended to multiple interval mapping [3] with multiple traits and incomplete phenotypic data. Third, mixed-model QTL mapping as recommended by [9] can now be applied to incomplete trait data as an alternative method for multi-trait QTL mapping. When multiple traits are actually different expressions of a single trait in different environments (locations or years), a mixed model allows treating environmental effect as a random and QTL effect as a fixed factor [39,40]. One of the advantages of the mixed model is in accommodating both balanced and unbalanced data structure.

In principle, our EM approach may be used to handle multi-trait QTL mapping with any proportion of missing phenotypic data. But if the data may not be MAR, as is especially likely when the missing proportion is large, a more prudent course of analysis is to find out why not, and work out an appropriate statistical method for QTL mapping.

The method we have presented requires more computing time than the conventional EM or ECM interval-mapping algorithm. There are two reasons for this. First, to obtain parameter estimates, the EM algorithm must be applied under both null and alternative hypotheses, because the trait data are missing in both cases. In contrast, conventional methods require EM iteration only under the alternative hypothesis. Second, our EM algorithm is used to complete both QTL genotype and phenotype in the case of ML-based QTL mapping, while the conventional method must complete only QTL genotype. The computing load increases with the proportion of missing data, but the extreme amounts of missing data we have simulated are unusual in real experiments.

## Methods

### Missing-data mechanism is ignored

Several kinds of "missingness" have been defined [37]. Under MAR, "missing at random", the probability of missing phenotypic data within any genotype class is unrelated to the phenotypic value. Either for MAR or the stronger assumption, MCAR or "missing completely at random" (missingness also independent of genotype), estimation methods need not model a missing-data mechanism. Either assumption seems reasonable in conventional mapping practice and is accommodated by the method described here.

### Multivariate regression with incomplete data

Consider the linear model

(1) **Y**_*n *× *m*_= **X**_*n *× *p *_**B**_*p *× *m *_+ **E**_*n *× *m*_,

where **Y **is a (*n *× *m*) response matrix with *n *the number of individuals and *m *the number of traits (or environments); **X **is a (*n *× *p*) design matrix with *p *predictors; **B **is a matrix of regression coefficients associated with **X**, **E **is an error matrix; and E_*i *_(*i *= 1, 2, ..., *n*) follows a multivariate normal distribution with means zero and variance-covariance matrix

(2) $$V=\left(\begin{array}{cccc} \sigma_{11}^{2} & \sigma_{12}^{2} & \cdots & \sigma_{1m}^{2}\\ \sigma_{21}^{2} & \sigma_{22}^{2} & \cdots & \sigma_{2m}^{2}\\ \vdots & \vdots & \ddots & \vdots \\ \sigma_{m1}^{2} & \sigma_{m2}^{2} & \cdots & \sigma_{mm}^{2} \end{array}\right)$$

For a random sample, the log likelihood of observations is given by

(3) $$\ell (B,V;Y)=-\frac{nm}{2}\ln(2\pi )-\frac{1}{2}\sum_{i=1}^{n}\ln|V|-\frac{1}{2}\sum_{i=1}^{n}{\left(y_{i}-x_{i}B\right)}^{\text{T}}V^{-1}(y_{i}-x_{i}B)$$

where **y**_*i *_is the response and **x**_*i *_the predictor vector of the *i*^th ^individual, **Y**^T ^= [**y**_1_, **y**_2_, ..., **y**_*n*_], and **X**^T ^= [**x**_1_, **x**_2_, ..., **x**_*n*_]. Parameters **B **and **V **are estimated by maximization of (3).

Suppose there are some missing entries in **y**_*i*_. The log likelihood of observations can be written as

(4) $$\ell (B,V;Y)=-\frac{nm}{2}\ln(2\pi )-\frac{1}{2}\sum_{i=1}^{n}\ln\left|V_{i}^{obs,obs}\right|-\frac{1}{2}\sum_{i=1}^{n}{\left(y_{i}^{obs}-x_{i}^{obs}b^{obs}\right)}^{\text{T}}{\left(V_{i}^{obs,obs}\right)}^{-1}(y_{i}^{obs}-x_{i}^{obs}b^{obs})$$

where $y_{i}^{obs}$ is the observed part of **y**_*i*_, $x_{i}^{obs}$ is the part of the predictor vector associated with $y_{i}^{obs}$, **b**^*obs *^contains the regression coefficients associated with $x_{i}^{obs}$, and $V_{i}^{obs,obs}$ is the submatrix of **V **representing the variance-covariance matrix of the traits for which the *i*^th ^individual has complete data. Since missing entries vary among individuals, the log likelihood is a logarithm sum of multivariate normal probabilities of varying dimensions.

Estimates of parameters **B **and **V **cannot be obtained by direct maximization of (4) with respect to the individual parameters. To estimate parameters in the presence of missing data we may apply the EM algorithm of [35]. For the *i*^th ^individual with some missing trait entries, we partition its trait **y**_*i*_, its mean **μ**_*i *_= **x**_*i*_**B**, and the variance-covariance matrix **V **as

(5) $$y_{i}=[y_{i}^{obs},y_{i}^{miss}],$$

where $y_{i}^{miss}$ is a vector composed of the missing trait data of individual *i*,

(6) $$\mu_{i}=[\mu_{i}^{obs},\mu_{i}^{miss}],$$

and

(7) $$V=\left[\begin{array}{cc} V_{i}^{obs,obs} & V_{i}^{obs,miss}\\ V_{i}^{miss,obs} & V_{i}^{miss,miss} \end{array}\right].$$

Given these partitions, the MLEs of parameters in model (1) are obtained as follows.

ALGORITHM 1: Starting with random initial values ${\hat{\theta }}^{\left(0\right)}=[{\hat{B}}^{\left(0\right)},{\hat{\mu }}^{\left(0\right)},{\hat{V}}^{\left(0\right)}]$, iterate the following two steps until convergence.

*E *step: use the following equation to predict missing trait data conditional on the observed trait data and variance-covariance matrix.

(8) $$E(y_{i}^{miss(k+1)}|y_{i}^{obs},\theta^{\left(k\right)})={\hat{\mu }}_{i}^{miss(k+1)}+(y_{i}^{obs}-{\hat{\mu }}_{i}^{obs(k+1)}){\left({\hat{V}}_{i}^{obs,miss}\right)}^{\left(k\right)}{\left({\hat{V}}_{i}^{obs,obs}\right)}^{-1(k)}.$$

Then reconstruct complete phenotypic matrix **y**_*i *_with the observed and predicted trait data (*k *indexes iterations):

(9) $$y_{i}^{\left(k+1\right)}=(y_{i}^{obs},y_{i}^{miss(k+1)}).$$

*M *step:

(10) $${\hat{B}}^{\left(k+1\right)}={\left(X^{\text{T}}X\right)}^{-1}X^{\text{T}}Y^{\left(k+1\right)},$$

(11) $${\hat{\mu }}^{\left(k+1\right)}=X{\hat{B}}^{\left(k+1\right)},$$

(12) $${\hat{V}}^{\left(k+1\right)}=\frac{{\left(Y^{\left(k+1\right)}-{\hat{\mu }}^{\left(k+1\right)}\right)}^{\text{T}}(Y^{\left(k+1\right)}-{\hat{\mu }}^{\left(k+1\right)})}{n}.$$

### Multi-trait QTL mapping with incomplete phenotypic data by regression

We now describe our multi-trait QTL mapping method with incomplete data. Though the method given is based on a recombinant inbred line (RIL) population, it is readily extended to other mating designs, as we show for the F_2_. According to the statistical model for multiple-trait analysis [9,10,19] based on complete phenotypic data, the model for incomplete phenotypic data is written as

(13) **Y**_*n *× *m *_= **z**_*n *× 1_**a**_(1 × *m*) _+ **x**_*n *× (*p*+1)_**b**_(*p*+1) × *m *_+ **E**_*n *× *m*_

where **Y **= [**y**_1_, **y**_2_,..., **y**_*n*_]' is a matrix of phenotypic data for *n *lines and *m *traits, and **y**_1_, **y**_2_,...,**y**_*n *_are 1 × *m *vectors composed of observations and missing trait data; **z **is a matrix of QTL genotypes represented as 2 for *QQ *and 0 for *qq*; **a **is a matrix of additive effects of a putative QTL at a tested position; **x **is a matrix of genotypes of *p *cofactor markers with the first column ones; **b **is a matrix of cofactor marker effects; and **E **is a matrix of residual errors *e*_*ij *_(*i *= 1, 2, ..., *n*; *j *= 1, 2,..., *m*), which are assumed to be correlated between traits and to follow a multivariate normal distribution with means zero and covariance matrix as in (2). Equation (13) is readily seen to be a variant of (1).

In this model, QTL genotype is replaced with its conditional expectation given flanking-marker genotypes [1,26]. Least-squares estimates of the parameters can then be obtained by multiple regression based on ALGORITHM 1. If considering a F_2 _population, we may instead use the model

(14) **Y**_*n *× *m *_= **z**_*n *× 1_**a**_1 × *m *_+ **w**_*n *× 1_**d**_1 × *m *_+ **x**_*n *× (*p*+1)_**b**_(*p*+1) × *m *_+ **E**_*n *× *m*_,

where **w **is a matrix of QTL genotypes represented as 1 for *Qq *and 0 for *qq *and *QQ*, and **d **is a matrix of dominance effects of a putative QTL at a tested position. Since w is unobservable, it is also replaced by its probability conditional on flanking markers.

### Multitrait QTL mapping with incomplete phenotypic data by ECM

Instead of replacing a missing QTL genotype with its expectation given flanking markers, ECM (expectation/conditional maximization) treats QTL genotype as missing data included in model (14) and estimates parameters at a QTL position by repeatedly updating the posterior probability of QTL genotype given both flanking-marker genotypes and phenotypes. Since we now have two types of missing data in model (14), QTL genotype and phenotype, we may extend the ECM method of [9] for multi-trait QTL mapping as follows:

ALGORITHM 2: Starting with initial values of parameters

(15) $$\theta^{\left(0\right)}=[{\hat{a}}^{\left(0\right)},{\hat{b}}^{\left(0\right)},{\hat{\mu }}^{\left(0\right)},{\hat{V}}^{\left(0\right)}],$$

iterate the following two steps until convergence (< 10^-7 ^change in log likelihood between two iterations).

*E *step:

(16) $$q_{1i}^{\left(k+1\right)}=\frac{p_{1i}f_{1}^{\left(k\right)}(y_{i}^{obs}|{\hat{\mu }}_{i,QQ}^{\left(k\right)},{\hat{V}}_{i}^{obs,obs(k)})}{p_{1i}f_{1}^{\left(k\right)}(y_{i}^{obs}|{\hat{\mu }}_{i,QQ}^{\left(k\right)},{\hat{V}}_{i}^{obs,obs(k)})+p_{2i}f_{2}^{\left(k\right)}(y_{i}^{obs}|{\hat{\mu }}_{i,qq}^{\left(k\right)},{\hat{V}}_{i}^{obs,obs(k)})},$$

(17) $$q_{2i}^{\left(k+1\right)}=\frac{p_{1i}f_{2}^{\left(k\right)}(y_{i}^{obs}|{\hat{\mu }}_{i,qq}^{\left(k\right)},{\hat{V}}_{i}^{obs,obs(k)})}{p_{1i}f_{1}^{\left(k\right)}(y_{i}^{obs}|{\hat{\mu }}_{i,QQ}^{\left(k\right)},{\hat{V}}_{i}^{obs,obs(k)})+p_{2i}f_{2}^{\left(k\right)}(y_{i}^{obs}|{\hat{\mu }}_{i,qq}^{\left(k\right)},{\hat{V}}_{i}^{obs,obs(k)})},$$

where *p*_1*i *_and *p*_2*i *_are the conditional probabilities of QTL genotypes *QQ *and *qq *given flanking markers and recombination distances [26], *f *the multivariate normal probability density function, and *q*_1*i *_and *q*_2*i *_the posterior probability of QTL genotypes given flanking markers and phenotypes [9].

(18) $$E(y_{i}^{miss(k+1)}|y_{i}^{obs},{\hat{\theta }}^{\left(k\right)})={\hat{\mu }}_{i,E}^{miss(k+1)}+(y_{i}^{obs}-{\hat{\mu }}_{i,E}^{obs(k+1)}){\hat{V}}_{i}^{obs,miss(k)}{\left({\hat{V}}_{i}^{obs,obs}\right)}^{-1(k)},$$

(19) $$y_{i}^{\left(k+1\right)}=(y_{i}^{obs},y_{i}^{miss(k+1)}).$$

*M *step:

(20) $${\hat{a}}^{\left(k+1\right)}=\frac{0.5q_{2}^{(k+1)\text{T}}(Y^{\left(k+1\right)}-x{\hat{b}}^{\left(k+1\right)})}{q_{2}^{\left(k+1)\text{T}\right)}l},$$

where **l **is a (*n *× 1) matrix of ones.

(21) $${\hat{b}}^{\left(k+1\right)}={(x^{\text{T}}x)}^{-1}x^{\text{T}}[Y^{\left(k+1\right)}-2q_{2}^{\left(k+1\right)}{\hat{a}}^{\left(k+1\right)}],$$

(22) $${\hat{\mu }}_{E}^{\left(k+1\right)}=x{\hat{b}}^{\left(k+1\right)}+2q_{2}^{\left(k+1\right)}{\hat{a}}^{\left(k+1\right)},$$

where ${\hat{\mu }}_{E}^{\left(k+1\right)}$ is the predicted phenotypic mean given QTL and cofactor markers,

(23) $${\hat{\mu }}_{QQ}^{\left(k+1\right)}={\hat{\mu }}_{E}^{\left(k+1\right)},$$

(24) $${\hat{\mu }}_{qq}^{\left(k+1\right)}=x{\hat{b}}^{\left(k+1\right)}$$

where ${\hat{\mu }}_{QQ}^{\left(k+1\right)}$ and ${\hat{\mu }}_{qq}^{\left(k+1\right)}$ are the predicted phenotypic means of QTL genotypes *QQ *and *qq *given cofactor markers, and

(25) $${\hat{V}}^{\left(k+1\right)}=\frac{{\left(Y^{\left(k+1\right)}-{\hat{\mu }}_{E}^{\left(k+1\right)}\right)}^{\text{T}}(Y^{\left(k+1\right)}-{\hat{\mu }}_{E}^{\left(k+1\right)})}{n}.$$

For a F_2 _population, we need to consider both additive **a **and dominance effects **d **in terms of model (14). In this case, the E-step is as in equations 16–19, and the M-step is as follows:

(26) $${\hat{a}}^{\left(k+1\right)}=\frac{0.5q_{2}^{(k+1)\text{T}}}{q_{2}^{(k+1)\text{T}}l}(Y^{\left(k+1\right)}-x{\hat{b}}^{\left(k+1\right)}),$$

(27) $${\hat{d}}^{\left(k+1\right)}=(\frac{q_{1}^{(k+1)\text{T}}}{q_{1}^{(k+1)\text{T}}l}-\frac{0.5q_{2}^{(k+1)\text{T}}}{q_{2}^{\left(k+1)\text{T}\right)}l})(Y^{\left(k+1\right)}-x{\hat{b}}^{\left(k+1\right)})$$

where **l **is a (*n *× 1) matrix of ones,

(28) $${\hat{b}}^{\left(k+1\right)}={(x^{\text{T}}x)}^{-1}x^{\text{T}}[Y^{\left(k+1\right)}-2q_{2}^{\left(k+1\right)}{\hat{a}}^{\left(k+1\right)}-q_{1}^{\left(k+1\right)}({\hat{a}}^{\left(k+1\right)}+{\hat{d}}^{\left(k+1\right)})],$$

(29) $${\hat{\mu }}_{E}^{\left(k+1\right)}=x{\hat{b}}^{\left(k+1\right)}+2q_{2}^{\left(k+1\right)}{\hat{a}}^{\left(k+1\right)}+q_{1}^{\left(k+1\right)}({\hat{a}}^{\left(k+1\right)}+{\hat{d}}^{\left(k+1\right)}),$$

where ${\hat{\mu }}_{E}^{\left(k+1\right)}$ is the predicted phenotypic mean given QTL and cofactor markers,

(30) $${\hat{\mu }}_{QQ}^{\left(k+1\right)}=x{\hat{b}}^{\left(k+1\right)}+2q_{2}^{\left(k+1\right)}{\hat{a}}^{\left(k+1\right)},$$

(31) $${\hat{\mu }}_{Qq}^{\left(k+1\right)}=x{\hat{b}}^{\left(k+1\right)}+q_{1}^{\left(k+1\right)}({\hat{a}}^{\left(k+1\right)}+{\hat{d}}^{\left(k+1\right)}),$$

(32) $${\hat{\mu }}_{qq}^{\left(k+1\right)}=x{\hat{b}}^{\left(k+1\right)},$$

where ${\hat{\mu }}_{QQ}^{\left(k+1\right)}$, ${\hat{\mu }}_{Qq}^{\left(k+1\right)}$ and ${\hat{\mu }}_{qq}^{\left(k+1\right)}$ are the predicted phenotypic means of QTL genotypes *QQ*, *Qq*, and *qq *given cofactor markers, and **V **is updated by (25).

In the ECM algorithm, incautious selection of initial parameter values may lead to convergence on local maxima. We used additive and dominance effects of 0, and the estimates of **μ **and **V **under the null hypothesis H_0_: *a *= 0, *d *= 0.

### Hypothesis tests for QTL effects with missing phenotypic data

Hypothesis tests for QTL main effects, pleiotropy effects and close linkage vs. pleiotropy are constructed according to [9] and can be tested by ALGORITHM 1 if regression is chosen or ALGORITHM 2 if the ECM method is used. As test statistic the likelihood ratio (LR) or its transformation to a logarithm-of-odds (LOD) are commonly used. For example, to test main QTL effects in a two-trait example, the hypotheses can be formulated as *H*_0_: *a*_1 _= 0, *a*_2 _= 0 and *H*_1_: at least one *a ≠ *0. For the regression method, parameters under H_0 _or H_1 _are estimated by ALGORITHM 1 (Equations 8–12) depending on whether or not QTL effects are included in model (13). If the ECM method is used, first these quantities are estimated under H_0 _by ALGORITHM 1 without inclusion of QTL effect and then those of the full model under H_1 _are obtained by ALGORITHM 2 (Equations 16–25 for RIL or 16–19, 26–32, and 25 for F_2_). Then the LR is obtained as *LR *= -2(ℓ_*reduced *_- ℓ_*full*_), where ℓ_*reduced *_is the log likelihood of the reduced model under H_0_, and ℓ_*full *_is that of the full model under H_1 _[4]. Both are calculated from (4) and a LOD score is calculated as *LR*/(2 ln 10).

### Simulations

To compare the properties of the EM method with those of casewise deletion (CaD), mean substitution (MS), conditional mean substitution (CMS) and complete data (CoD), we performed simulation experiments. RIL populations of size 100, 200 and 300 were generated based on a 300-cM chromosome with 31 evenly spaced markers. For CMS, missing data were replaced with their conditional expectations calculated by regression of each trait on the other(s). Three pleiotropic QTLs controlling two traits were simulated at cM positions 53, 182, and 258 with effects listed in Table 2.

**Table 2:** QTL effects and variances for two traits used for simulation of multi-trait QTL mapping.

**Parameter**	**QTL**	**Trait**	
		
		**1**	**2**
**QTL effect**	**1**	2.3	2
	**2**	-2.1	-1.8
	**3**	1.7	1.6
**QTL variance**	**1**	5.3 (8.4%)	4.0 (8.2%)
	**2**	4.4 (7.0%)	3.2 (6.6%)
	**3**	2. 9 (4.6%)	2.6 (5.2%)
**Total genetic variance**		12.6 (20%)	9.8 (20%)
**Environmental variance**		50.0 (80%)	39.2 (80%)
**Phenotypic variance**		62.6 (100%)	49.0 (100%)

Trait values of each line were calculated as the sum of QTL effects plus a random vector of environmental effects with means zero and variance given in Table 2. Then a specified proportion (0.05, 0.10, 0.20, or 0.40) of values for each trait independently was set to missing. Lines lacking data for both traits were dropped. Analyses were performed on 500 replicates.

In the QTL analyses, the calculation interval (step size) used was 1 cM. Cofactor markers for each trait were selected by forward stepwise regression at a significance level of 0.01 and combined for multi-trait analysis. Cofactors lying within 10 cM of a QTL testing position were dropped from the model.

Though the LR at each test position asymptotically follows a chi-square distribution with degrees of freedom determined by the corresponding hypothesis test, an acceptance threshold applying over all test positions must be found. Genomewide LOD thresholds of 3.71, 3.54 and 3.43 for *n *= 100, 200, and 300 at significance level 0.05 were calculated from 5000 simulations with no missing data under the null hypothesis of no QTL [20]. When sample size or heritability is relatively small, the effect of a QTL may extend to adjacent intervals due to sampling error. Rather than including heritability as an experimental parameter for investigation, we chose the cautious expedient of declaring a QTL if a LOD peak higher than threshold was found within the interval containing the simulated QTL and the intervals on either side of the QTL interval. Power of QTL detection was calculated as the number of correctly declared ("true positive") QTLs divided by the number of actual QTLs simulated, while specificity was calculated as the number of true positive QTLs divided by the total number declared.

### Real data analysis

We applied the EM method to a population of 325 doubled-haploid lines (unpublished data) tested for rice sheath-blight disease in field and greenhouse studies and genotyped with 114 codominant markers. The traits we chose for analysis were sheath-blight score and heading date measured in Stuttgart, Arkansas in 2006 and correlated at *r *= -0.57. The preliminary SIM and CIM analyses indicated that a QTL on chromosome 12 influenced both traits, and suggested a multi-trait analysis.

Since there were no missing trait data, we generated a new sample with 10% randomly missing phenotype scores. Genomewide LOD thresholds 3.73, 3.77, 3.90, 3.80, and 3.83 for EM, CoD, CaD, MS, and CMS at significance level 0.05 were calculated from 1000 permutations, based on shuffling the phenotypic records for both traits at once in order to preserve their correlation structure.

## Authors' contributions

ZG conceived the problem, developed the statistical approach, and drafted the manuscript. JCN guided the design and conduct of the experimental study and co-wrote the manuscript. Both authors read and approved the final manuscript.

## References

[B1] Haley CS, Knott SA (1992). A simple regression method for mapping quantitative trait loci in line crosses using flanking markers. Heredity.

[B2] Jansen RC (1993). Interval mapping of multiple quantitative trait loci. Genetics.

[B3] Kao C-H, Zeng Z-B, Teasdale RD (1999). Multiple interval mapping for quantitative trait loci. Genetics.

[B4] Lander ES, Botstein D (1989). Mapping Mendelian factors underlying quantitative traits using RFLP linkage maps. Genetics.

[B5] Satagopan JM, Yandell BS, Newton MA, Osborn TC (1996). A Bayesian approach to detect quantitative trait loci using Markov chain Monte Carlo. Genetics.

[B6] Wang H, Zhang Y-M, Li X, Masinde GL, Mohan S, Baylink DJ, Xu S (2005). Bayesian shrinkage estimation of quantitative trait loci parameters. Genetics.

[B7] Yi N, Xu S (2001). Bayesian mapping of quantitative trait loci under complicated mating designs. Genetics.

[B8] Zeng Z-B (1994). Precision mapping of quantitative trait loci. Genetics.

[B9] Jiang C, Zeng Z-B (1995). Multiple trait analysis of genetic mapping for quantitative trait loci. Genetics.

[B10] Korol AB, Ronin YI, Kirzhner VM (1995). Interval mapping of quantitative trait loci employing correlated trait complexes. Genetics.

[B11] Ma CX, Casella G, Wu RL (2002). Functional mapping of quantitative trait loci underlying the character process: A theoretical framework. Genetics.

[B12] Allison DB, Thiel B, St Jean P, Elston RC, Infante MC, Schork NJ (1998). Multiple phenotype modeling in gene-mapping studies of quantitative traits: Power advantages. Am J Hum Genet.

[B13] Evans DM (2002). The power of multivariate quantitative-trait loci linkage analysis is influenced by the correlation between variables. Am J Hum Genet.

[B14] Kučerová J, Lund MS, Sorensen P, Sahana G, Guldbrandtsen B, Nielsen VH, Thomsen B, Bendixen C (2006). Multitrait quantitative trait loci mapping for milk production traits in Danish Holstein cattle. J Dairy Sci.

[B15] Neuschl C, Brockmann GA, Knott SA (2007). Multiple-trait QTL mapping for body and organ weights in a cross between NMRI8 and DBA/2 mice. Genet Res.

[B16] Thomasen J (2008). Quantitative trait loci affecting calving traits in Danish Holstein cattle. J Dairy Sci.

[B17] Malosetti M (2008). A multi-trait multi-environment QTL mixed model with an application to drought and nitrogen stress trials in maize (*Zea mays *L.). Euphytica.

[B18] Calinski T, Kaczmarek Z, Krajewski P, Frova C, Sari-Gorla M (2000). A multivariate approach to the problem of QTL localization. Heredity.

[B19] Hackett CA, Meyer RC, Thomas WTB (2001). Multi-trait QTL mapping in barley using multivariate regression. Genet Res.

[B20] Knott SA, Haley CS (2000). Multitrait least squares for quantitative trait loci detection. Genetics.

[B21] Korol AB, Ronin YI, Nevo E, Hayes PM (1998). Multi-interval mapping of correlated trait complexes: simulation analysis and evidence from barley. Heredity.

[B22] Xu S (1995). A comment on the simple regression method for interval mapping. Genetics.

[B23] Kao C-H (2000). On the differences between maximum likelihood and regression interval mapping in the analyis of quantitative trait loci. Genetics.

[B24] Mangin B, Thoquet P, Grimsley N (1998). Pleiotropic QTL analysis. Biometrics.

[B25] Weller JI, Wiggans GR, VanRaden PM, Ron M (1996). Application of a canonical transformation to detection of quantitative trait loci with the aid of genetic markers in a multi-trait experiment. Theor Appl Genet.

[B26] Jiang C, Zeng Z-B (1997). Mapping quantitative trait loci with dominant and missing markers in various crosses from two inbred lines. Genetica.

[B27] Sen S, Churchill GA (2001). A statistical framework for quantitative trait mapping. Genetics.

[B28] Sillanpaa MJ, Arjas E (1999). Bayesian mapping of multiple quantitative trait loci from incomplete outbred offspring data. Genetics.

[B29] Sillanpää MJ, Arjas E (1998). Bayesian mapping of multiple quantitative trait loci from incomplete inbred line cross data. Genetics.

[B30] Allison PD (2002). Missing Data.

[B31] Rubin DB (1987). Multiple Imputation for Nonresponse in Surveys.

[B32] Rubin DB (1996). Multiple imputation after 18+ years. Journal of the American Statistical Association.

[B33] Dempster AP, Laird NM, Rubin DB (1977). Maximum likelihood from incomplete data via the EM algorithm. J Royal Stat Soc B.

[B34] Niu T, Ding AA, Kreutz R, Lindpaintner K (2005). An expectation-maximization-likelihood-ratio test for handling missing data: application in experimental crosses. Genetics.

[B35] Little RJA, Rubin DB (2001). Statistical Analysis with Missing Data.

[B36] Liu JF, Liu YJ, Liu XG, Deng HW (2007). Bayesian mapping of quantitative trait loci for multiple complex traits with the use of variance components. Am J Hum Genet.

[B37] Rubin DB (1976). Inference and missing data. Biometrika.

[B38] Piepho HP (2001). A quick method for computing approximate thresholds for quantitative trait loci detection. Genetics.

[B39] Piepho H-P (2000). A mixed-model approach to mapping quantitative trait loci in barley on the basis of multiple environment data. Genetics.

[B40] Wang DL, Zhu J, Li ZK, Paterson AH (1999). Mapping QTLs with epistatic effects and QTL × environment interactions by mixed linear model approaches. Theor Appl Genet.

